# P-881. Outpatient Antibiotic Prescribing Patterns for Urinary Tract Infections at a Single Veterans Affair Medical Center ​

**DOI:** 10.1093/ofid/ofaf695.1089

**Published:** 2026-01-11

**Authors:** Tina H Dao, Brian Porter, Daniel Lee, Dana Douglass, Jessica G Bennett

**Affiliations:** University of Tennessee Health Science Center College of Medicine, Memphis, TN, Memphis, Tennessee; University of Tennessee Health Science Center, Memphis, Tennessee; University of Tennessee Health Science Center College of Medicine, Memphis, TN; South Texas Veterans Health Care System, San Antonio, Texas; VAMC Memphis, Germantown, Tennessee

## Abstract

**Background:**

Urinary tract infection (UTI) is among the top 3 diagnoses resulting in antibiotic prescriptions in outpatient adult visits. Studies have demonstrated appropriate antibiotic choice and duration are selected ∼50% of the time. In this project, we aim to evaluate local prescribing practices and adherence to facility guidance for UTI treatment.

**Figure:**
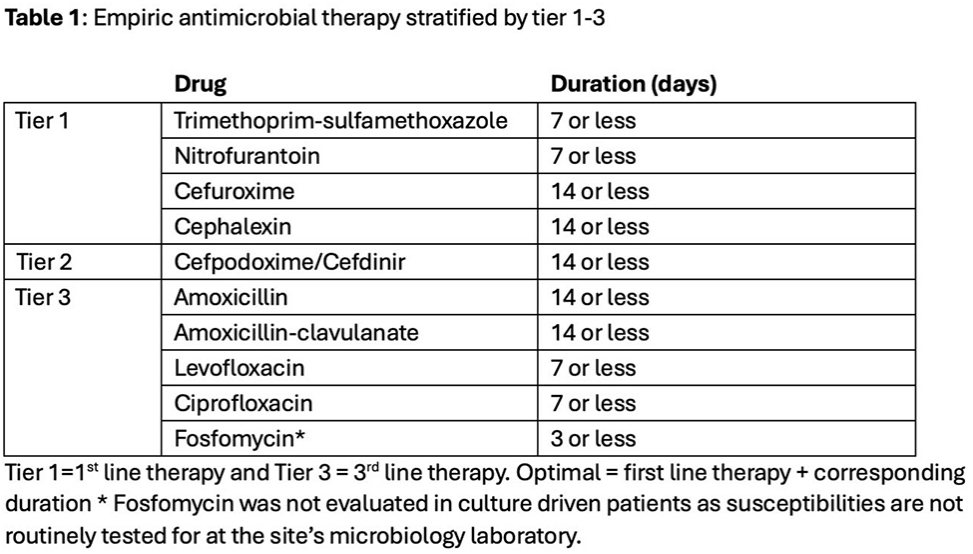

**Figure:**
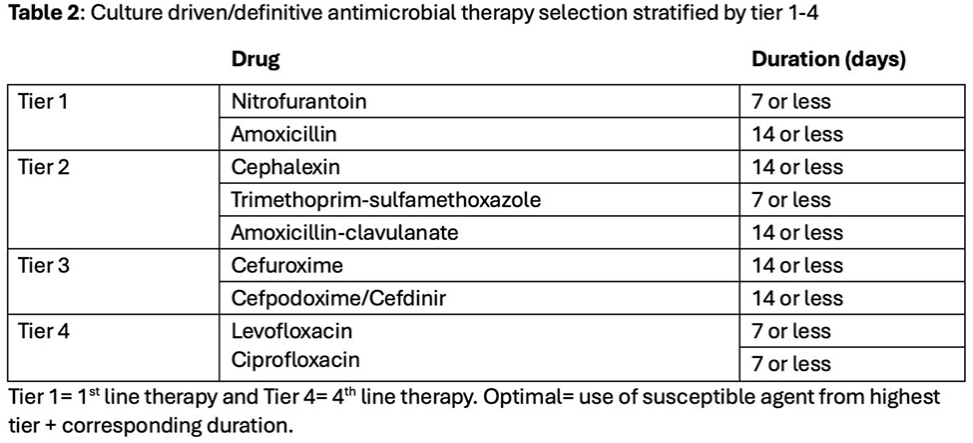

**Methods:**

This retrospective single center study identified outpatient antibiotics written for UTI from 7/1/2024-9/30/2024. Demographic information, antibiotic choice, duration, prescriber, and location of prescription were extracted from the Corporate Data Warehouse. Symptoms of UTI, culture and sensitivity reports were obtained via chart review. Antibiotics were stratified into tiers based on facility guidelines and antibiogram (Tables 1 & 2). Antibiotics were rated as optimal, sub-optimal or inadequate. Optimal was defined as highest tier drug (empiric) or highest tier drug susceptible to culture with recommended duration. Sub-optimal was lower tier drug and/or longer than recommended duration. Inadequate was defined as treatment of ASB or selection of resistant antibiotic for culture-driven prescription. Allergies and renal function were also considered. Data was analyzed using descriptive statistics.

**Figure:**
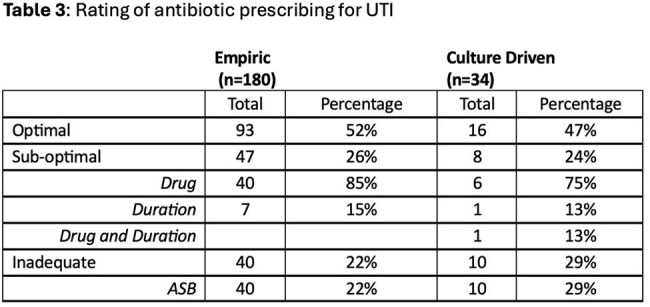

**Figure:**
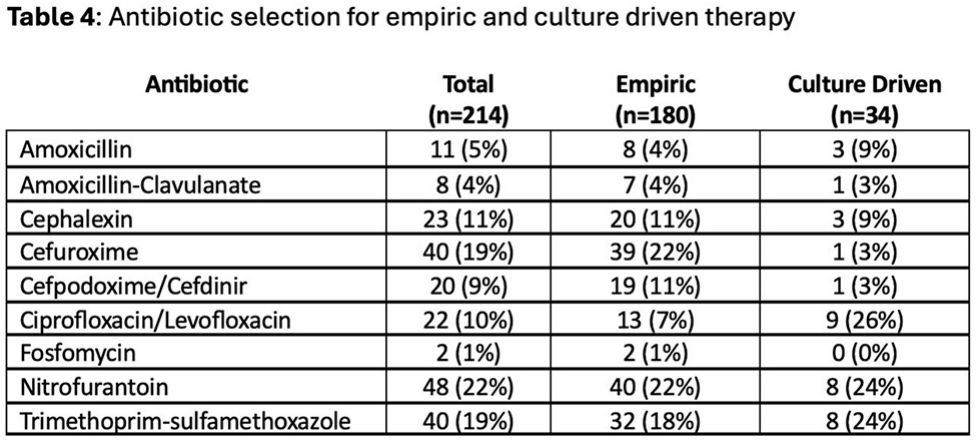

**Results:**

269 prescriptions were identified and 214 were analyzed. The population was largely older males (146 (80%), avg age 66.6 (26-95)). Most prescriptions were from the Emergency Department (133, 62%) and were empirically prescribed (180, 84%). Approximately half of the prescriptions were optimal (Table 3). 79 (44%) of empirically prescribed antibiotics had cultures drawn. Of these, 61 (77%) did not require a change in antibiotics, 13 (16%) had a culture with bacteria resistant to selected antibiotics and 5 (6%) were prescribed for asymptomatic bacteriuria. Trimethoprim/sulfamethoxazole, nitrofurantoin and cefuroxime were the most prescribed (Table 4). The average duration was 7.18 days (range 3-30 days).

**Conclusion:**

This review shows our facility follows previously published trends with ∼50% of antibiotics written for UTI adherent to local practice guidelines. This was due largely to antibiotic choice and provides baseline data to guide antibiotic stewardship interventions.

**Disclosures:**

All Authors: No reported disclosures

